# Associations between Cytokines and Metabolic Parameters in Patients with Schizophrenia - Pilot Study

**DOI:** 10.1192/j.eurpsy.2026.12159

**Published:** 2026-07-31

**Authors:** K. Krysta, B. Trędzbor, E. Martyniak, J. Fojcik, K. Piekarska-Bugiel, A. Koźmin-Burzyńska, M. Krzystanek

**Affiliations:** 1 Department of Rehabilitation Psychiatry; 2 Department of Psychiatry, Department of Neurology, Faculty of Health Sciences in Katowice, Medical University of Silesia, Katowice, Poland

## Abstract

**Introduction:**

Metabolic disturbances are common in patients with schizophrenia and are increasingly linked to immune dysregulation. Cytokines such as interleukin-10 (IL-10), an anti-inflammatory mediator, may serve as biomarkers reflecting the interplay between inflammation and metabolic imbalance. Previous studies have shown inconsistent results regarding cytokine profiles in schizophrenia, but recent evidence suggests that IL-10 expression may increase in states of insulin resistance and dyslipidemia (Kamei et al. *Genes* 2022; 13(2):294).

Metabolic disturbances are common in patients with schizophrenia and are increasingly linked to immune dysregulation. Cytokines such as interleukin-10 (IL-10), an anti-inflammatory mediator, may serve as biomarkers reflecting the interplay between inflammation and metabolic imbalance. Previous studies have shown inconsistent results regarding cytokine profiles in schizophrenia, but recent evidence suggests that IL-10 expression may increase in states of insulin resistance and dyslipidemia (Kamei et al. *Genes* 2022; 13(2):294).

**Objectives:**

This study aimed to assess the relationship between pro- and anti-inflammatory cytokines (IL-1β, IL-6, IL-8, IL-10, IL-12, TNF-α) and metabolic indicators (glucose, total cholesterol, triglycerides, insulin, HOMA-IR) in patients with schizophrenia.

**Methods:**

A cross-sectional analysis was conducted among 21 adult patients (mean age 31.4 ± 9.2 years; 12 males, 9 females) diagnosed with schizophrenia according to ICD-10 criteria. Blood samples were analyzed for cytokines (IL-1β, IL-6, IL-8, IL-10, IL-12, TNF-α) using immunoassay techniques. Metabolic parameters (glucose, total cholesterol, triglycerides, insulin, HOMA-IR) were determined with standard biochemical methods. Pearson’s correlation coefficients were used to assess associations between cytokines and metabolic measures (significance level p < 0.05).

**Results:**

Significant positive correlations were found between IL-10 and lipid parameters: total cholesterol (r = 0.47, p = 0.036) and triglycerides (r = 0.73, p < 0.001). No significant associations were observed between IL-10 and glucose, insulin, or HOMA-IR. Other cytokines (IL-1β, IL-6, IL-8, IL-12, TNF-α) showed no significant correlations with metabolic markers.

**Conclusions:**

In this cross-sectional study of patients with schizophrenia, IL-10 demonstrated a positive association with dyslipidemia, while other cytokines showed no significant metabolic relationships. These findings suggest that IL-10 may act as an immuno-metabolic biomarker, reflecting compensatory anti-inflammatory activity in response to metabolic stress. The results align with previous reports linking IL-10 up-regulation to lipid abnormalities in insulin-resistant states (Tarkowski et al. Front Psychiatry 2021; 12:536257; Chen et al. BMC Psychiatry 2024; 24:6054).

**Disclosure of Interest:**

None Declared

